# Prophylactic use of levosimendan in pediatric patients undergoing cardiac surgery: the jury is still out

**DOI:** 10.1186/s13054-020-2812-z

**Published:** 2020-03-23

**Authors:** Xavier Beretta-Piccoli, Dominique Biarent, David De Bels, Patrick M. Honore, Sébastien Redant

**Affiliations:** 1 ICU Department, Hôpital Universitaire des Enfants Reine Fabiola HUDERF, Université Libre de Bruxelles, ULB, Av J.J. Crocq 15, 1020 Brussels, Belgium; 2 ICU Department, Centre Hospitalier Universitaire Brugmann-BrugmannUniversity Hospital, Place Van Gehuchtenplein,4, 1020 Brussels, Belgium

The study by Wang et al. on levosimendan used in prophylaxis of low cardiac output syndrome (LCOS) post-pediatric cardiac surgery is the first study comparing levosimendan versus placebo. The authors did not show an impact on mortality or on the occurrence of LCOS [1]. However, it is very interesting to see that in the multivariate regression analysis, levosimendan had an impact on the onset of LCOS (OR 0.38 and *p* = 0.037). We are not surprised by the absence of an impact on mortality given the small number of patients. In a Cochrane review, Hummel et al. estimated that the number of pediatric patients required to prove the superiority of levosimendan for outcome mortality was 652 [2]. We wonder how levosimendan could prevent LCOS. LCOS in a heart with good preoperative function is linked to inadequate myocardial protection during aortic clamping, poorly performed cardioplegia, myocardial reperfusion lesions, ventriculotomy, preoperative arrhythmias, activation of inflammatory cascades and complement, and alterations of pulmonary and systemic vascular resistances [3, 4]. Because of its intrinsic mode of action as a calcium sensitizer, we could expect levosimendan to improve cardiac function after surgery but probably not to prevent intraoperative events. The study of Wang et al. showed the safety of using levosimendan in a population with a preoperative cardiac index of 3.3 ml/min/m^2^ and low-risk adjustment in congenital heart surgery (RACHS) [5]. This may lead to another question: are those patients sick enough to show a benefit from levosimendan? The study therefore opens the door to others comparing levosimendan to placebo in more severe pediatric patients with higher RACHS, preoperative decompensation, and/or low cardiac output with a cardiac index < 2.2 L min/m^2^.

## Authors’ response

Anbiao Wang, Chaomei Cui, and Qi Tan

We appreciate the comments of Dr. Sébastien Redant to our recent article regarding the prophylactic use of levosimendan in pediatric patients undergoing cardiac surgery.

The hypothesis that levosimendan is a safe and effective inodilator when used in cardiac surgery could not be supported until now [6]. Levosimendan was found to have no impact on mortality or low cardiac output syndrome (LCOS); however, a multivariate regression analysis indicated that levosimendan administration was a risk factor associated with LCOS in our patient cohort.

In the study conducted by Hummel et al., their total 864-patient sample size was calculated based on the incidence of mortality [7]. Different from their study, we elected to focus on LCOS rather than mortality as the primary outcome. This decision was informed by the work of Ricci et al., who found that LCOS occurred in 37% of patients in their levosimendan group and in 61% of controls [8]. Based on the more accurate LCOS diagnostic criteria by Cardiac Index (CI), we anticipated lower LCOS incidence rates of 45% and 25% in the control and levosimendan groups, respectively. We agree that a larger sample size would be preferable, and we are still in the process of collecting clinical data in an effort to extend this study.

Levosimendan was not able to effectively prevent intraoperative events. However, our data indicated that CI significantly deteriorated at 2 h post-surgery (2.60 ± 0.69 and 2.64 ± 0.78, respectively, in the levosimendan and placebo groups). This suggests that the unique pathophysiological changes that occur after surgery are stressful and lead to a high risk of LCOS during this critical period. Considering the inotropic effect, lusitropic effect, and protection against ischemia/reperfusion up to 81 h of levosimendan and its metabolite [9, 10], prophylactic use of levosimendan after cardiac surgery may be a promising and reasonable approach to reducing the risk of subsequent LCOS development. In our experience, levosimendan should not be treated as an emergency medication in the same manner as adrenaline or norepinephrine. As such, once LCOS symptoms including tachycardia or hypotension occurred, levosimendan administration was not recommended.

With respect to concerns regarding whether these patients were sick enough for levosimendan administration, there is currently no consensus as to what a reasonable level of indication is in this context. In addition to referring to risk adjustment in congenital heart surgery (RACHS), we additionally considered lower age, low body weight, and non-cardiac congenital structural abnormalities such as pulmonary arterial hypertension when making our clinical determinations.

In summary, while further research is needed, we are confident in the results of our randomized study and feel that these findings should be an impetus for additional well-designed and focused studies of this topic.

## Data Availability

Not applicable.

## Abbreviations

LCOS: Low cardiac output syndrome
RACHS: Risk adjustment in congenital heart surgery
